# Studies on Pitting Corrosion of Al-Cu-Li Alloys Part II: Breakdown Potential and Pit Initiation

**DOI:** 10.3390/ma12111786

**Published:** 2019-06-02

**Authors:** Elmira Ghanbari, Alireza Saatchi, Xiaowei Lei, Digby D. Macdonald

**Affiliations:** Department of Materials Science and Engineering, University of California at Berkeley, Berkeley, CA 94720, USA; elmira.ghanbari@berkeley.edu (E.G.); alireza.saatchi@berkeley.edu (A.S.); leixw1987@gmail.com (X.L.)

**Keywords:** Al-Cu-Li alloys, point defect model, Al-Li alloys, passivity breakdown

## Abstract

Prediction of the accumulated pitting corrosion damage in aluminum-lithium (Al-Li) is of great importance due to the wide application of these alloys in the aerospace industry. The Point Defect Model (PDM) is arguably one of the most well-developed techniques for evaluating the electrochemical behavior of passive metals. In this paper, the passivity breakdown and pitting corrosion performance of AA 2098-T851 was investigated using the PDM with the potentiodynamic polarization (PDP) technique in NaCl solutions at different scan rates, Cl^−^ concentrations and pH. Both the PDM predictions and experiments reveal linear relationships between the critical breakdown potential (*E_c_*) of the alloy and various independent variables, such as $a_{Cl^{-}}$ and pH. Optimization of the PDM of the near-normally distributed *E_c_* as measured in at least 20 replicate experiments under each set of conditions, allowing for the estimation of some of the critical parameters on barrier layer generation and dissolution, such as the critical areal concentration of condensed cation vacancies (*ξ*) at the metal/barrier layer interface and the mean diffusivity of the cation vacancy in the barrier layer (*D*). With these values obtained—using PDM optimization—in one set of conditions, the *E_c_* distribution can be predicted for any other set of conditions (combinations of $a_{{\mathrm{Cl}}^{-}}$, pH and T). The PDM predictions and experimental observations in this work are in close agreement.

## 1. Introduction

While composites have been introduced as alternative lightweight materials for aerospace applications, aluminum (Al) alloys have been extensively studied and have evolved to remain a competitive choice due to their low cost, natural self-passivation properties, high strength-to-weight ratio, and corrosion resistance [1]. To this day, Al alloys still account for about 60% of the weight of most transport aircrafts.

Among different alloying elements for Al alloys, Li has been of particular interest due to the low density, strength, and excellent fatigue crack resistance of Al-Li alloys [1,2,3]. As aluminum is active in nature, once the passive film is subjected to breakdown, the initiation and development of corrosion pits could be very different than with other passive metals/alloys. Moreover, lithium is an active alkali metal, so the presence of lithium will make the formation and breakdown of passive films on aluminum alloys even more complicated [2,3,4,5,6,7,8]. This explains the importance of the study of the corrosion performance of the Al-Li alloys, which is one of the main focuses of this work.

The third generation Al-Li alloys, compared to previous Al-Li alloys series, have improved properties in corrosion resistance, yield strength, isotropy, short-transverse ductility, short-transverse fracture toughness, and thermal stability [9]. The pitting behavior of the third generation Al-Li alloys are of great importance with significant safety implications for the aircraft industry where life prediction of the airframe components is of crucial importance. This work is focused on pitting corrosion, considering the fact that evolution from pitting to intergranular corrosion (IGC) in high strength Al alloys is quite common [2,3,4,5,6,10]. Stress corrosion and corrosion fatigue cracks tend to nucleate at pits, and IGC usually starts at the bottom of pits and subsequently grows into a large network [7]. Zhang et al. reported that IGC and exfoliation corrosion (EC) of the third generation Al-Li alloys strongly depend on the composition, heat treatment, and grain orientation of these alloys [8]. The lower Li content of third-generation compared to second-generation Al-Li alloys results in significant susceptibility to IGC [11,12,13]. Additionally, the copper content in the third-generation Al-Cu-Li in saline environments increases the IGC susceptibility of these alloys [9].

Unlike the vast literature devoted to the corrosion behavior and its mechanism of other series of Al alloys, there is still a need for further analyses of the corrosion mechanisms of the third-generation Al-Cu-Li alloys. So far, most of the efforts on the subject have been focused on the T1 phase (Al_2_CuLi) as the main reason for the localized corrosion susceptibility of Al-Cu-Li alloys, due to its rapid anodic and cathodic reaction kinetics, which is based on the classical concepts in the literature for other types of Al alloys. Some researchers have considered the anodic dissolution of T1 precipitates at the grain boundaries in Al-Cu-Li alloys since the presence of the active Li in the T1 phase was considered to increase the susceptibility of these alloys to IGC [14,15,16,17,18,19,20]. Alternatively, according to other researchers, the T1 phase may also act as a cathode with the selective dissolution of Li [7] which is similar to the nanoscale dealloying of Mg in an S phase Al_2_CuMg that leaves behind Cu rich precipitate [21,22]. The dealloying process of T1 intermetallic compounds and its composition modification due to the minor alloying additions in the grain boundaries of third-generation Al-Li alloys has not been thoroughly studied.

Based on the PDM, all different metallurgical features at the metal/barrier layer interface (m/bl), such as grain boundaries, matrix/inclusions or precipitates interface, dislocations that project through the barrier layer, etc., could be potential pit nucleation sites. This is due to the presence of the maximal structural disorders at these regions that result in a high diffusivity of cation vacancies (noting that, based on the PDM, cation vacancies migrate not diffuse because of the high electric field, ε = 3 × 10^6^ V/cm and that the migration flux is proportional to D) through the barrier layer, with the cation vacancies being produced at the barrier layer/solution interface (bl/s) interface by cation injection [23].

In the present work, the corrosion behaviors of AA2029-T8, AA2060-T8 and AA2098-T851 with different Li contents were investigated in NaCl-containing solutions to simulate environments such as in atmospheric flight in marine environments. Two of the alloys examined in this study are third-generation Al-Cu-Li alloys, AA 2060-T8 (S2) and AA 2098-T851 (S3). Another Al alloy, AA2029-T8 (S1), which did not contain Li, was also chosen for assessing the effect of Li. The feature that these alloys have in common is that they were all intended to replace 2024-T3 with a superior combination of high yield strength, improved fracture toughness, and corrosion resistance for high damage tolerant aerospace applications, such as in fuselage and pressure cabin skin. However, these alloys are prone to localized corrosion. This is due to their heterogeneous microstructure, which may result in galvanic corrosion between Al and its inclusions [24,25]. Therefore, prediction of the service life of these alloys as determined by localized corrosion (pitting), requires the development of a deterministic model for the passivity breakdown (critical breakdown) potential and the induction time and for predicting the distributions in these quantities. In paper Part I [26], the microstructure and pitting behavior of the three alloys were studied. Using different electron microscopy techniques, it was shown that the microstructures and especially the precipitates of the S2 and S3 alloys, which contain lithium, are different from the lithium-free Alloy (S1). Specifically, needle-like precipitates that could be of the T1 phase (Al_2_CuLi) were present in these alloys, which seemed to have a finer structure with increased lithium content in the S3 [27,28,29,30,31]. Furthermore, potentiodynamic tests showed that the S3 alloy had the widest passive region and lowest steady-state current density. Finally, immersion tests coupled with in situ observations followed by pit depth measurements revealed that although the S3 alloy showed the fastest pitting with a higher number of pits, pits in this alloy were significantly shallower than those in the S1 and the S2 alloys.

Presently, in Part II, more insight into breakdown potential and pitting initiation is provided using further potentiodynamic polarization testing and surface characterization followed by analytical modeling. Among all analytical methods for modeling the passivation of pure metals and alloys, the PDM is arguably one of the most well-developed theoretical models [32,33,34,35,36,37] and, to our knowledge, it has shown no conflict with the experimental observations. As will be discussed in more detail in the next part of this series, the PDM is based on a set of reactions describing the generation and annihilation of point defects (metal interstitials and cation and oxygen vacancies) and interactions between the crystallographic (point) defects in the metal and the barrier layer of the passive film with and without aggressive anions such as [Cl^−^] in the surrounding environment. Additionally, in this model, the significant influence of pH and temperature on the kinetics of these reactions is also considered [36,37].

Of the three alloys examined in this work, AA2098-T851 with higher Li content, which was shown in Part I to have the best pitting resistance, was considered for further analysis of the *E_c_* based on the PDM. The ultimate objective of this paper was the measurement of the distribution in the *E_c_* for the Alloy S3. The *E_c_* is a near-normally-distributed parameter that determines the susceptibility of the metal to pitting corrosion. This parameter can be measured experimentally using the PDP [38,39].

The PDM predicts relationships for calculating the statistical cumulative probability distribution functions of the critical breakdown potential and induction time as a function of the activity of the aggressive anion and pH on the distribution in *E_c_*. Both experimental observations and PDM predictions confirm that *E_c_* decreases with increasing the chloride concentration in the solution and with decreasing pH. The analytical relationships between *E_c_* and independent experimental variables, such as [Cl^−^], pH and PDP scan rate, provided by the PDM, allows the estimation of some characteristic PDM parameters, such as *α*, *β* and *ξ*. These constants can be further used in exploring passivity breakdown over much wider ranges of conditions. To the best of our knowledge, using such an approach for the investigation and prediction of passivity breakdown has not been done for Al-Cu-Li alloys, which is the ultimate goal of this work. This study will be coupled with studies of passivation and film growth kinetics of Al-Cu-Li alloys, which will be discussed in the next part of this series.

## 2. Materials and Methods

### 2.1. Materials

In this work, the corrosion behavior of AA2029-T8 (S1), AA2060-T8 (S2) and AA2098-T851 (S3) using electrochemical measurements were investigated. The nominal chemical compositions of the aluminum alloys used in this study are shown in Table 1. It is noted that the lithium content is the most notable compositional variable for the three of Al alloys. Additionally, the magnesium and zinc contents are slightly different among these alloys. The notation T8 stands for solution heat treated, cold worked, then artificially aged, and T851 means solution heat treated, stress-relieved by stretching, then artificially aged.

### 2.2. Passive Film Characterization

Before electrochemical analyses, the passive film and its composition on the three types of Al alloys was analyzed by X-Ray Photo-electron Spectrometry (XPS) using a Axis Ultra DLD XPS instrument (Kratos Analytical Ltd, Shimadzu Corporation, Tokyo, Japan), and an FEI Quanta 3D FEG scanning electron microscope (SEM) (FEI, Hillsboro, OR, USA) coupled with an Oxford Instruments Energy Dispersive Spectroscopy (EDS) for chemical composition analysis. For all experiments, the exposed surfaces were abraded using SiC sandpapers of successively finer grit from 400 to 1200, and then rinsed sequentially with acetone, ethanol, and double distilled water and finally dried with N_2_ gas. The passive film was prepared in 0.01 M NaCl solution with 0.1 M NaHCO_3_, and CO_2_ was purged into the solution during the immersion of samples in the solution, which was done for 2 h at room temperature (25 °C). After immersion, the specimens were gently cleaned with ethanol and naturally dried in a desiccator filled with N_2_ gas to reduce the risk of modification of the film prior to examination.

For the XPS analyses, the monochromatic radiation method (Al Ka, 1486.6 eV) was applied for the excitation of photoelectrons. The XPS spatial resolution was 5 μm, and the energetic resolution was 0.45 eV (Ag 3 d 5/2). The XPS data were fitted by using the XPS Peak software (version 4.1).

The SEM voltage for imaging the passive film and its precipitates was 10 kV, and samples were not coated prior to SEM observations.

### 2.3. Electrochemical Measurements

For electrochemical studies, a copper wire was glued to the Al alloy coupons with a conductive silver paste and the coupons were then mounted in epoxy resin with 0.35 cm^2^ of exposed area. The corrosion performance of the aluminum alloys was investigated in NaCl solution in a CO_2_ atmosphere prepared using analytical reagent grade chemicals. Three important components of the atmosphere that establish the corrosion potential, the pH, and the breakdown potential are O_2_, CO_2_, and Cl^−^, respectively. Even in the case of space vehicles, the vehicle is exposed to a corrosive atmosphere during flight and during pre-flight on the launch pad, in the assembly building, etc. Under some operating conditions, such as flight at high altitude, the oxygen level falls drastically, which is one of the reasons for performing the experiments in O_2_− free environments. Another reason is avoiding the complications imposed by the cathodic partial reaction of oxygen reduction.

Furthermore, under any set of conditions (T, [O_2_], pH) it is not [O_2_], per se, that is important, it is the corrosion potential, which can be readily calculated using the Mixed Potential Model (MPM) by employing the data obtained in this work and elsewhere. Therefore, unless there is a specific interaction of carbonate or bicarbonate with the barrier layer, the results obtained in this work are transferable to some other system, provided that no specific anion/barrier layer is present and that [Cl^−^] and pH are known. In this paper, during electrochemical analyses, the solution pH was maintained to be constant (6.7) throughout the experiments by using the 0.1 M NaHCO_3_ buffer in the presence of CO_2_ gas. Additionally, all experiments were conducted at ambient temperature (25 °C).

**Figure 1:**
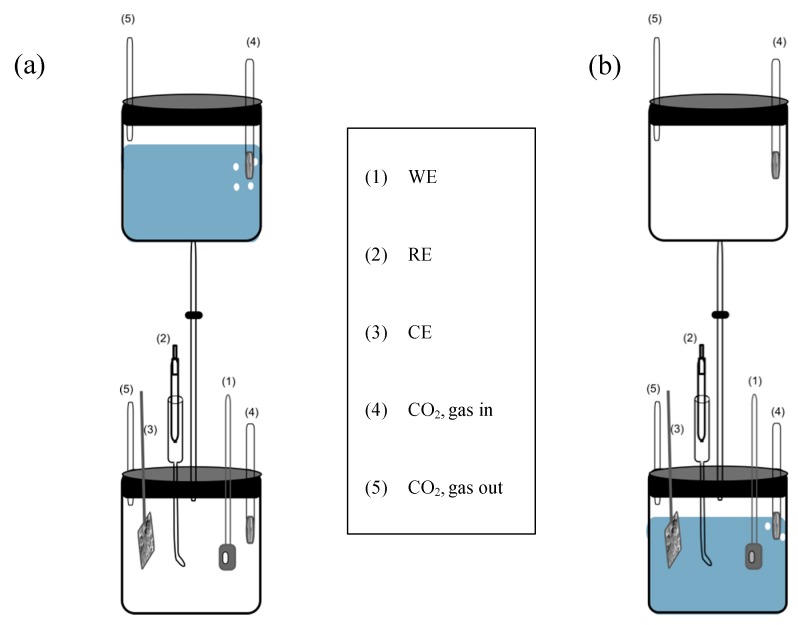
The schematic of the electrochemical cell, (**a**) prior to the experiment and (**b**) during the experiment.

To expel oxygen from the system, a 200-mL double cell, which is shown in Figure 1, was used. Before an experiment, the electrolyte in the first cell was purged with high purity CO_2_ gas for at least one hour. Meanwhile, the newly polished sample was placed in the second cell with no solution and also deaerated with CO_2_ (Figure 1a). After 1-hour of deaeration, the solution in the first cell was transferred to the second cell containing the sample (Figure 1b). Additionally, to eliminate any possibility of the oxygen entering the cell, high purity (99.9 %) CO_2_ purging was continued throughout the experiment.

Electrochemical experiments were carried out in a conventional three-electrode electrochemical cell with an Al alloy sample as the working electrode (WE), a saturated calomel electrode (SCE) as a reference electrode (RE) which was kept in a Luggin capillary and, finally, a platinum mesh that served as the counter electrode (CE). All electrochemical measurements were performed using a Gamry Electrochemical Measurement System (PC3).

Prior to the experiments, samples were cathodically polarized at −1 V_SCE_ for 5 min and then left at open circuit potential (OCP) for 15 min. The potentiodynamic polarization (PDP) curves of three alloys were recorded at least ten times at a scanning rate of 0.167 mV/s in a 0.01 M NaCl concentration with NaHCO_3_ buffer solution in atmospheric CO_2_ condition.

Additionally, only for the Specimen (S3), in order to obtain the statistical distribution of the critical breakdown potential at different chloride ion concentrations (0.001, 0.005, 0.01, 0.05, 0.1, 0.5 and 1 M), a set of 20 PDP at a 0.166 mV/s scan rate were conducted at each [Cl^−^]. Moreover, the influence of the sweep rate on the passivity breakdown potential was studied by performing PDP at different scan rates (0.166, 1, 5 and 10 mV/s) at constant [Cl^−^] = 0.1 M on Alloy (S3). Finally, to investigate the relationship between *E_c_* and the solution pH at constant [Cl^−^] = 0.1 M and scan rate = 0.166 mV/s, PDP experiments were performed at three different pH values of 4.6, 5.6 and 6.7 corresponding to different buffer concentrations 0.001 M, 0.005 M and 0.1 M NaHCO_3_, respectively, on Specimen (S3).

## 3. Results

### 3.1. XPS Analysis of the Passive Film

**Figure 2:**
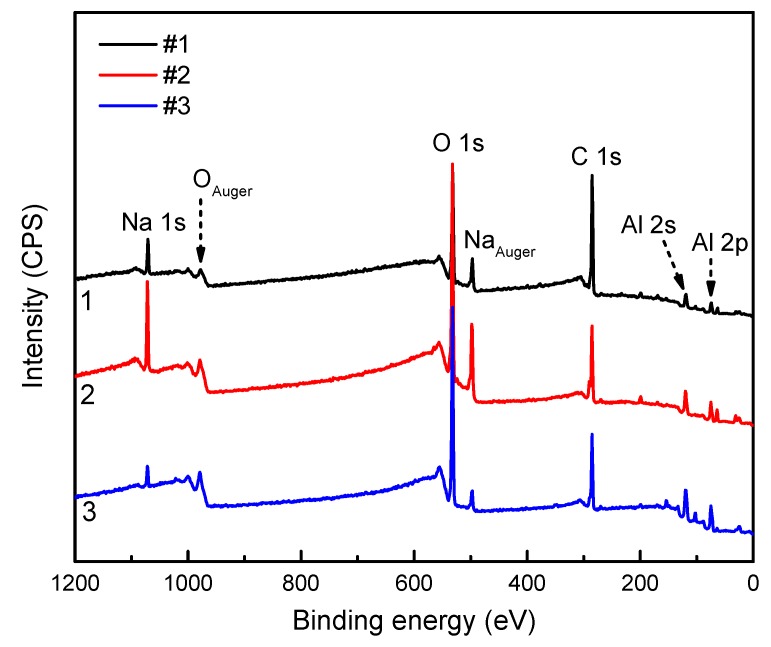
The large scale X-Ray Photo-electron Spectrometry (XPS) spectra of the three Al alloys, AA2029-T8 (#1), AA 2060-T8 (#2) and AA 2098-T851 (#3).

**Figure 3:**
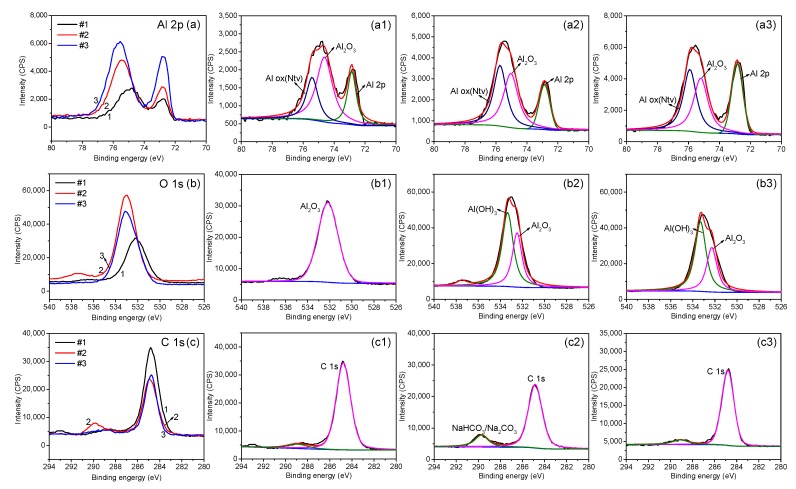
The XPS spectra of Al 2p (**a**), O 1s (**b**), and C 1s (**c**) for the three Al alloys. (**a1**,**b1**,**c1**): AA2029-T8 (S1); (**a2**,**b2**,**c2**): AA2060-T8 (S2); (**a3**,**b3**,**c3**): AA2098-T851 (S3).

Figure 2 shows the large scale XPS spectra of the three aluminum alloys. Despite the difference in chemical compositions and the microstructures of the alloy substrates, the passive film displays very similar composition, including the elements Al, O, C and Na. The presence of Na and C is because the corrosion media was comprised of NaCl and NaHCO_3_ and was purged with CO_2_. For a detailed comparison, the spectra of Al 2p and O 1s were analyzed, as shown in Figure 3. It can be seen that the passive film on the three specimens mainly contain Al_2_O_3_ and Al_ox_, where the Al_ox_ represents complex oxides that may be attributed to the air exposure during specimen transfer for XPS characterizations. Additionally, Al(OH)_3_ was found on the lithium-containing Specimens (S2 and S3), suggesting the presence of a hydroxide outer layer [Al(OH)_3_] on these two alloys.

### 3.2. Precipitates, Particles and Pit Nucleation

**Figure 4:**
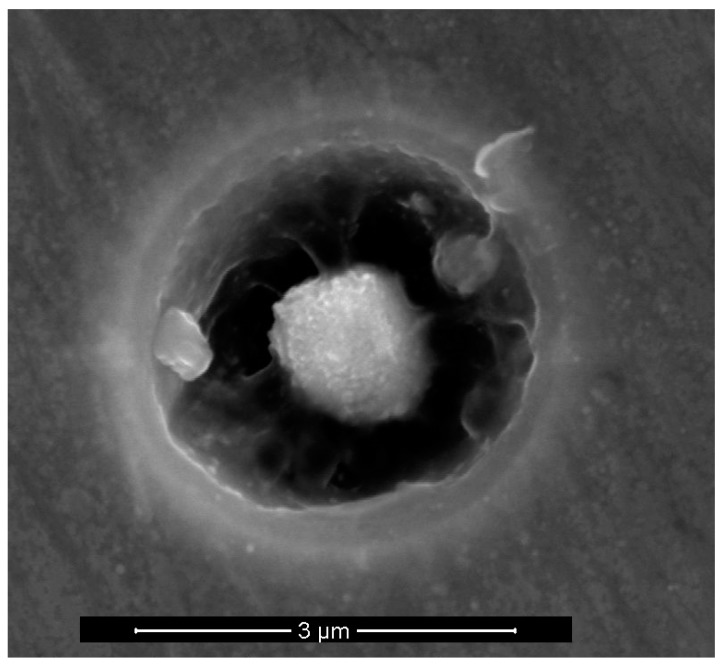
Scanning electron microscope (SEM) micrographs of early stages of pitting that nucleates at a particle from AA2098-T851 (S3).

**Figure 5:**
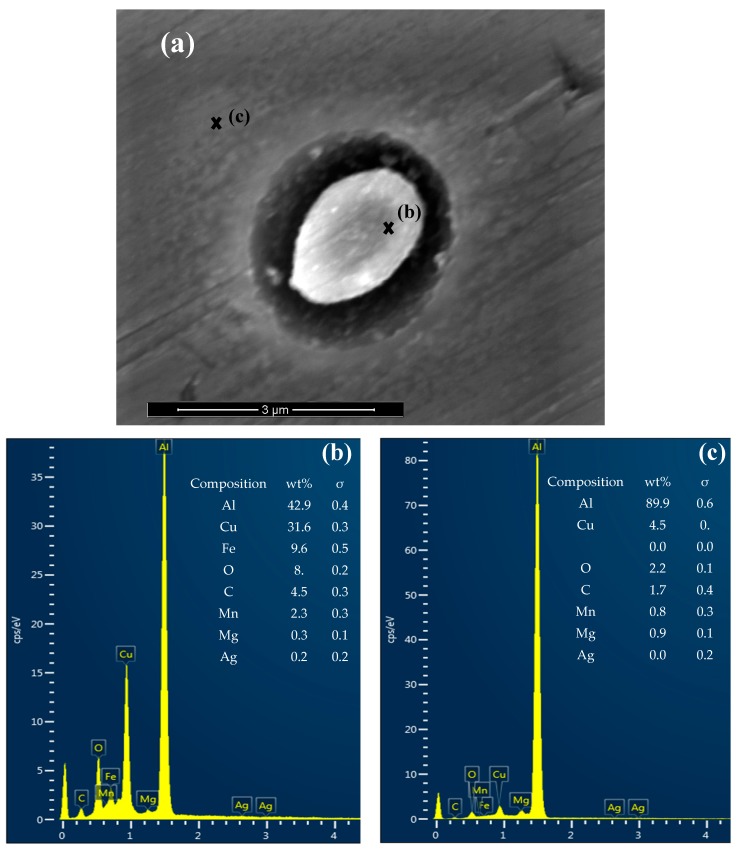
The SEM micrograph (**a**) and Energy Dispersive Spectroscopy (EDS) analysis (**b**,**c**) of a particle in AA2029-T8 (S1).

SEM and EDS analyses were performed on the three alloys after 2 h of immersion in 0.01 M NaCl solution with 0.1 M NaHCO_3_ and a CO_2_ atmosphere. Particles of different sizes and shapes were found that also had different chemical compositions. A frequent observation was the nucleation of pits at and surrounding the particles, as can be seen in Figure 4. Such occurrences are anticipated according to the literature [40], and as also discussed previously [26]. As far as the chemical composition of the particles is concerned, Li cannot be detected with EDS, and only the larger particles could be probed with reasonable accuracy. Figure 5 shows a SEM micrograph along with the EDS spectra of a typical particle observed in the samples. These particles were Cu rich and also contained some Fe. According to the literature and the approximate composition given by the EDS analyses, the particles such as the one shown in Figure 5a, could be composed of Al_2_CuM phase (*θ’*), or Al_2_Cu for the case of Alloy S1, where M could be one or more alloying elements, such as Li or Mg [40,41]. Others also reported the presence of Fe in the particles in the structures of similar alloys, which was attributed to the inaccuracy of the EDS measurement or the existence of iron impurities in the particle [40]. However, since Fe was consistently found in samples similar to that shown in Figure 5 and especially in the larger particles, other intermetallic phases in the Al-Cu-Fe system could also be possible. Further characterization is needed to describe and identify these phases.

### 3.3. Potentiodynamic Polarization

**Figure 6:**
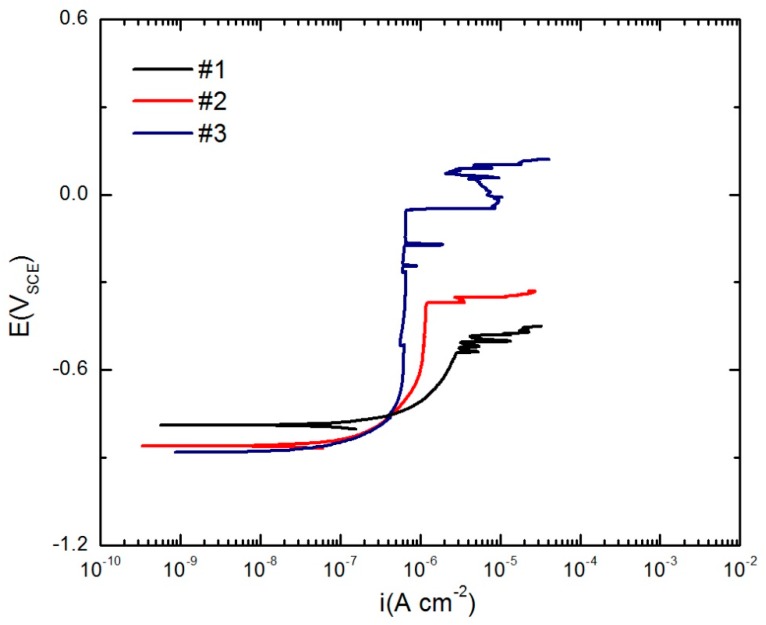
The potentiodynamic polarization curves of AA2029-T8 (S1), AA2060-T8 (S2) and AA2098-T851 (S3) in 0.01 M NaCl in NaHCO_3_ buffer solution in a CO_2_ atmosphere at room temperature. AA2029-T8 (#1), AA 2060-T8 (#2) and AA 2098-T851 (#3).

The corrosion performances of the Al Alloys S1, S2, and S3 were analyzed by a potentiodynamic polarization measurement, and the results are presented in Figure 6. It is noted that for each specimen, the potentiodynamic test was repeated ten times. For illustration purposes in Figure 6, the curve chosen for each of the samples is the one that had the closest values of corrosion potential (*E*_corr_), critical breakdown potential (*E*_c_), and passive current density ($i_{ss}^{bd}$) to the average of those values obtained for that sample. As can be seen in Figure 6, the *E*_c_ of Specimens S1 and S2 are close (S2 being slightly higher). As was also seen in similar experiments, but in a different buffer solution in Paper I, Figure 6 shows that the alloy with the highest Li content (Specimen S3) has the highest *E_c_* (most noble); suggesting that it has the best pitting resistance among these alloys.

This effect of lithium on the breakdown potential might be explained by the neutralization of pH in the depth of a growing nano-pit as a result of the reaction of H^+^ with electrons that are generated by the dissolution of lithium, but this explanation is problematic because neutralization could only occur after the passivity breakdown has taken place when the pit nucleus is open to the solution. Alternatively, it has been suggested that the reaction of lithium with water (Li + H_2_O → Li^+^ + 1/2H_2_ + OH^−^) might raise the pH of the solution that penetrates into the blister which, in turn, is expected to lower the rate of hydrogen evolution within the blister, resulting in a lower rate of pressurization of the blister and hence in a higher *E_c_*, as predicted by the PDM and found experimentally (Figure 6).

Henceforth, in this work, the Alloy S3 (AA2098-T851), which exhibits superior pitting resistance, was used in the study and modeling of the effects of the experimental variables on pitting corrosion.

### 3.4. Critical Breakdown Potential of AA2098-T851

**Figure 7:**
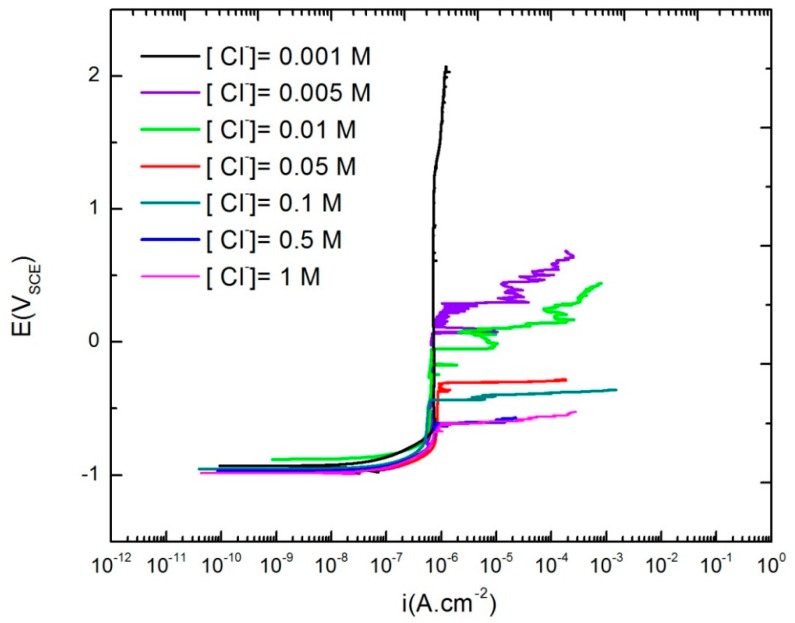
The potentiodynamic polarization curves of AA2098-T851 (S3) at different [Cl^−^] in NaHCO_3_ buffer solutions in a CO_2_ atmosphere at room temperature.

The critical breakdown potential is a near-normally distributed parameter [42,43,44,45]. To investigate the influence of chloride ion concentration [Cl^−^] on *E_c_* of AA2098-T851 (S3), a set of twenty replicate potentiodynamic polarization experiments were performed at a voltage scan rate of 0.166 mV/s for different [Cl^−^]. Figure 7 shows the general form of the polarization curves at different chloride concentrations. The plots in Figure 7 show the typical passive behavior, a wide flat passive region that followed by an increase in the current density at *E_c_*. Additionally, in some cases, metastable pitting, as indicated by the current spikes in the passive region appeared [46,47,48,49,50].

As predicted by the PDM and as reported by other authors, Figure 7 shows that a decrease of [Cl^−^] increases *E_c_* [39,42,44,45]. In Figure 7, we see that *E_c_* increases (becomes more positive) from −0.63 V_SCE_ for [Cl^−^] = 1 M to 0.29 V_SCE_ for [Cl^−^] = 0.005 M. Additionally, a further increase of [Cl^−^] from 0.5 M to 1 M did not change the *E_c_* values. This shows that the 0.5 M NaCl is close to the saturation point for the passivity breakdown of the samples in the NaHCO_3_ buffer solution in a CO_2_ atmosphere. At this point essentially, all of the available surface oxygen vacancies are occupied by chloride ions so that the further addition of Cl^−^ to the solution has no further impact on the generation of cation vacancies at the barrier layer/solution (bl/s) interface and hence upon *E_c_*. Additionally, Figure 7 shows that 0.001 M NaCl is too dilute to result in the formation of any localized corrosion in the AA2098-T851 in 0.1 M NaHCO_3_ buffer solution in a 1 atm CO_2_ atmosphere since there was no evidence of metastable pitting or breakdown in the passive region. Another feature to be noted in this figure is that for all [Cl^−^], the passive current density immediately before the passivity breakdown, ($i_{ss}^{bd}$), is almost constant and is equal to 10^−6^ A.cm^−2^, which is in agreement with the PDM hypothesis. According to the PDM, the absorption of aggressive anions into surface oxygen vacancies, which results in cation vacancy generation via cation abstraction, only affects the transmission (migration) of cation vacancies through the barrier layer. This issue will be discussed in detail in Part III of this publication series. Therefore, absorption of the aggressive anions into the surface oxygen vacancies is the fundamental cause of the breakdown of the barrier layer [51].

### 3.5. Passivity Breakdown of AA2098-T851

**Figure 8:**
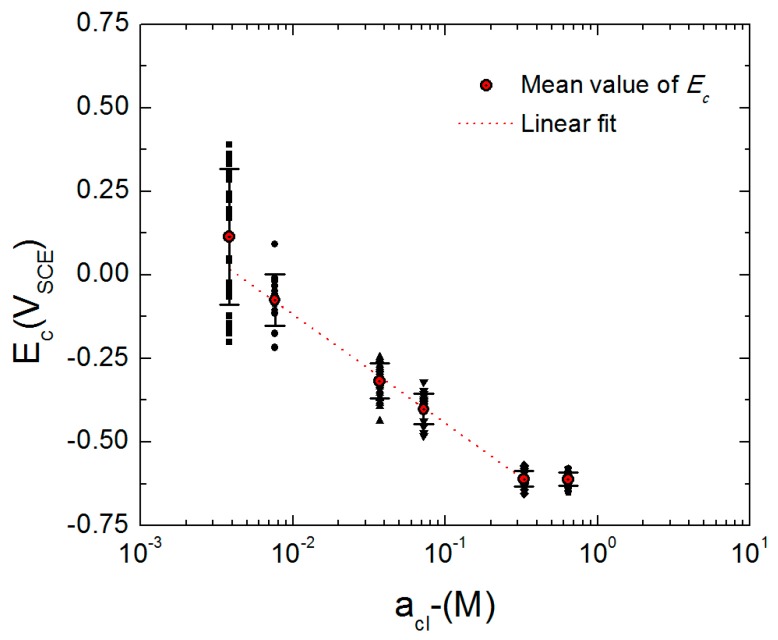
The mean value of the *E_c_* and its standard deviation for AA2098-T851 (S3) in 0.1 M NaHCO_3_ buffer solution in a CO_2_ atmosphere at T = 25 °C as a function of $\log(a_{{\mathrm{Cl}}^{-}}$) [53].

Figure 8 shows the mean values of the critical passivity breakdown potential of AA2098-T851 as a function of chloride ion activity ($a_{Cl^{-}}$) with the activity being estimated from the mean molar ionic activity coefficient ($\gamma_{\pm }$) of Cl^−^ in NaCl + 0.1 M NaHCO_3_ buffer solution in a CO_2_ atmosphere [52] and from the chloride concentration. This figure shows a linear decrease in *E_c_* with increasing $a_{Cl^{-}}$, which is also predicted by the PDM.

Additionally, as shown in Figure 8, at higher $a_{Cl^{-}}$, the standard deviation value for the distribution of *E_c_* became smaller. A similar observation was also made by Macdonald et al. for carbon steel in NaCl + saturated Ca(OH)_2_ solution [54].

**Figure 9:**
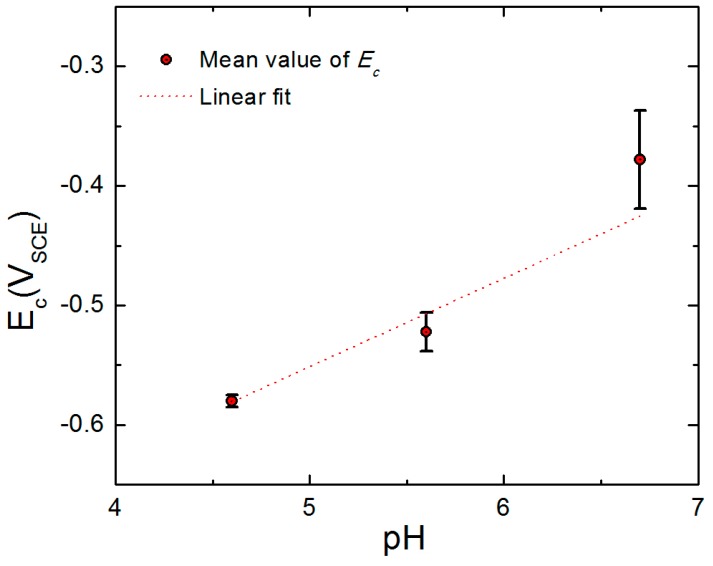
The *E_c_* as a function of pH for AA2098-T851 (S3) in 0.1 NaCl + NaHCO_3_ buffer solution in a CO_2_ atmosphere at 25 °C.

Figure 9 displays the variation of *E_c_* for AA2098-T851 as a function of the solution pH in 0.1 M NaCl with different concentrations of NaHCO_3_ buffer solution in a CO_2_ atmosphere. This figure shows that the breakdown potential linearly increases with increasing pH, which is in agreement with the prediction of the PDM and with observations made by other authors on different systems [23,55,56,57].

**Figure 10:**
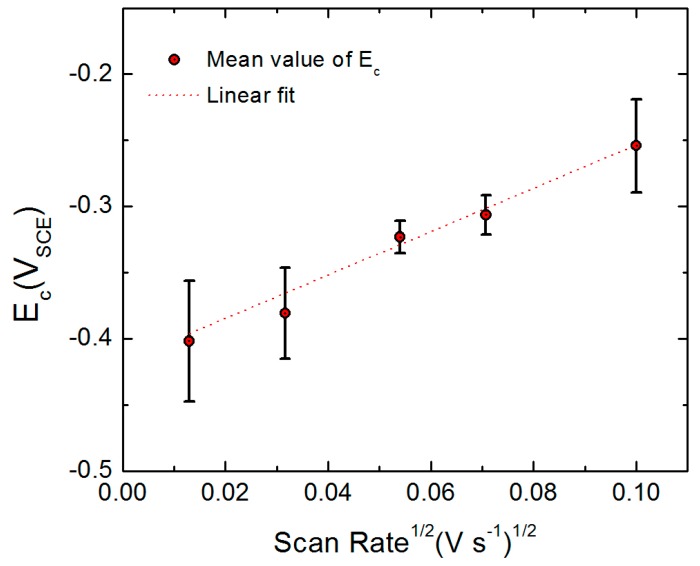
The *E_c_* as a function of voltage scan rate (*ν*) for AA2098-T851 (S3) in 0.1 M NaCl + 0.1 M NaHCO_3_ buffer solution in a CO_2_ atmosphere at 25 °C [53].

Figure 10 shows the *E_c_* of the AA2098-T851 as a function of the square root of potential scan rate (*ν*^1/2^) in 0.1 M NaCl + 0.1 M NaHCO_3_ buffer solution in a CO_2_ atmosphere. This figure shows that *E_c_* increases linearly with *ν*^1/2^ in potentiodynamic polarization [58,59,60,61,62,63,64,65]. This observation is also in agreement with the prediction of the PDM for the passivity breakdown. According to the PDM, the collapse of the barrier layer occurs by the condensation of cation vacancies at the m/bl interface followed by the dissolution of the “cap” (remnants of the barrier layer) over the condensate [23,66]. An increase of ν decreases the time for cation vacancy condensation at the m/bl interface, and as a result, a higher potential must be achieved to induce passivity breakdown. Additionally, both the PDM and the experiment reveal that the gradient of *E_c_* versus *ν*^1/2^ is independent of the chloride ion concentration [58,62,65]. Therefore, it is justified to only investigate the effect of *ν* on *E_c_* at a single chloride concentration (0.1 M).

## 4. Discussion

### 4.1. Passivity Breakdown of AA2098-T851 Based on the PDM

According to the PDM, *E_c_* is attributed to the attainment of critical conditions that result in cation vacancy condensation at the m/bl interface at the breakdown sites and concomitant dissolution of the barrier layer [36,37]. The PDM postulates that the passivity breakdown could only result from cessation of growth of the barrier layer into the substrate metal; otherwise if the film dissolved at the barrier layer/ solution interface to form a depression (a pit), the film would respond to the redistribution of potential by simply growing faster into the substrate metal and the “pit” would remain passive. The PDM suggests that the growth of the barrier layer into the metal ceases because cation vacancy condensation effectively separates the barrier layer from the substrate at that location [23]. To achieve the passivity breakdown condition, it is required that $\left(J_{ca}-J_{m}\right)\left(t-\tau \right))\geq \xi$, where *J_ca_* is the flux of migrating cation vacancies through the barrier layer, *J_m_* is the annihilation rate of cation vacancies at the m/bl interface, τ is the dissolution time of the cap over the vacancy condensate, and ξ is the areal (#/cm^2^) concentration of condensed cation sites at the m/bl interface. Note that as confirmed experimentally and predicted theoretically, the electric field strength (results in migration of the cation vacancies through the barrier layer) is independent of the applied voltage, but the rate of the cation ($M_{s}^{\delta +}$) ejection reaction at the bl/s interface, $M_{M}\to M_{s}^{\delta +}+V_{M}^{\chi \prime }+\left(\delta -\chi \right)e^{\prime }$, where $V_{M}^{\chi \prime }$ is cation vacancy on the metal sublattice and $M_{M}$ is metal cation on the metal lattice, is only weakly dependent upon the applied potential. On the other hand, the cation vacancy concentration at the barrier layer/solution interface (*C_bl/__s_*) increases strongly with increasing applied potential and increasing [Cl^−^]. The flux of migrating cation vacancies is given by $J_{ca}=z\gamma DC_{bl/s}\varepsilon$, where z is the electrical charge, $\gamma =\frac{F}{RT}$ and *D* is the cation vacancy diffusivity. Since the values of *z*, γ, *D*, ε are constants and are potential-independent, the increase in *J_ca_* to achieve the critical breakdown condition at the m/bl interface for cation vacancy condensation is due to the increase in *C_bl/__s_*. Please note that the *J_m_* value depends on the self-diffusion coefficient of the metal in the substrate metal and also the properties of the barrier layer that are potential-independent [23].

The Point Defect Model considers the effect of point defect formation and annihilation at the barrier layer boundaries and the dissolution of the barrier layer at the bl/s interface, and specifically accounts for the overall effects of microstructural defects, such as inclusions, as was qualitatively characterized in the previous sections. Passivity breakdown is considered to occur at the locations in the bl having high structural disorder, where the cation vacancy diffusivity is assumed to be highest and the applied potential-dependent flux of cation vacancy at the m/bl interface is higher than the rate of cation vacancy annihilation via cation injection from the metal at the same location [23,67]. At that point (potential, *E_c_*), vacancy condensation occurs and continues around the periphery of the blister. The initial condensation point may be nanoscopic, but the radius of the blister increases with the square root of time and quickly grows to a micron size, as observed by Bargeron and Givens and others [68,69,70,71,72,73,74], with the size being determined by the rate of dissolution and the mechanical integrity of the cap. Thus, at some point during this expansion, the blister (remnants of the dissolving barrier layer) fractures and allows the electrolyte to penetrate into the blister, resulting in the emission of gas (H_2_, H_2_S, NH_3_ and CH_4_, corresponding to oxide, sulfide, nitride, and carbide precipitates, respectively), as reported by Bargeron and Givens) [69,70,71]. The role of the gas is to pressurize the blister, which induces a fracture to produce the breakdown event. This is consistent with Luo’s observation that *E_c_* becomes more negative as hydrogen is injected into the backside of a thin metal membrane in a Devanathan cell [75], although they did not interpret their data in terms of that concept.

Regarding the unique behavior of the microstructure of the alloy, it is noted that PDM does not take the effect of the microstructure directly into account, except that the breakdown sites are postulated to be regions in the barrier layer that are characterized by high structural disorder, such as the points of intersection between the barrier layer and precipitates. Breakdown at the points of intersection between precipitates and the passive film has been reported in numerous studies [76,77,78,79,80]. According to the PDM, these sites are postulated to be characterized by high cation vacancy diffusivity and the population of sites is envisioned to be normally distributed in this parameter. As will be shown in this paper, the PDM accounts for the experimental distributions for the alloy studied, as it has done so for many other alloys (including carbon steel, stainless steel, and copper) [54,81,82].

In this paper, some of the PDM parameters, such as *α* and *β*, based, respectively, on the linear relationship between the critical breakdown potential and the chloride ion activity and pH, were calculated. Additionally, using the PDM, the correlation between *E_c_* and the square root of potential sweep rate (*ν*^1/2^) was used to estimate the critical cation vacancy concentration (ξ) at the m/bl interface. Finally, using these characteristic findings and optimization of the PDM on the distribution function for *E_c_* of AA2098-T851 in a chloride solution, the mean diffusivity of cation vacancy ($\bar{D}$) for this alloy was obtained.

According to the PDM, the linear dependence of *E_c_* on the logarithm of the aggressive anion activity ($a_{Cl^{-}}$) in the aqueous solution and also on the pH of the solution as observed in Figure 8 and Figure 9 are shown in Equation (1) [58]. It is important to note that *E_c_* is near-normally distributed, as discussed below, so that the apparent scatter in *E_c_* at each chloride activity is not due to random error and experimental uncertainty, but is an important physico-electrochemical property of the system.

(1) $$E_{c}=\frac{4.606RT}{\chi \alpha F}log\left(\frac{b}{D}\right)-\frac{2.303RT}{\alpha F}log\left(a_{Cl^{-}}\right)$$

where

(2) $$b=\frac{RTJ_{m}\Omega }{F\varepsilon N_{v}}exp\left(\frac{\left(\frac{\chi }{2}\right)\Delta G_{A}^{0}-\left(\frac{\chi }{2}\right)F\varphi_{f/s}^{0}+\Delta G_{s}^{0}-\left(\frac{\chi }{2}\right)\beta FpH}{RT}\right)$$

and *R* is the gas constant (8.314 J mol^−1^ K^−1^), *T* is the Kelvin temperature (K), *χ* is the stoichiometry of the oxide (MO_χ/2_), *α* is the polarizability constant that correlates the potential drop across the bl/s interface with the applied potential (*E_app_*), *D* is the diffusivity of the cation vacancy in the barrier layer (cm^2^ s^−1^), *J_m_* is the annihilation rate of cation vacancies at the m/bl interface, *F* is Faraday’s constant (96487 C mol^−1^), *ɛ* is the electric field strength within the barrier layer (V cm^−1^), *Ω* is the volume per mole of cations in the film (cm^3^ mol^−1^), *N_v_* is the Avogadro’s number (6.023 × 10^23^), *β* is the constant that relates the potential drop across the bl/s interface to the pH, $\Delta G_{A}^{0}$ is the standard Gibbs energy change due to the absorption reaction between Cl^−^ and Vö (J mol^−1^), $\Delta G_{s}^{0}$ is the standard Gibbs energy change for the Schottky pair reaction or for chloride-induced cation extraction from the bl/s interface (J mol^−1^), and $\varphi_{f/s}^{0}$ is the potential drop at the bl/s at pH = 0 and *E_app_* = 0. Equation (2) can be simplified by substituting all of the energy-related parameters into Equation (3) as [58]

(3) $$\omega =\left(\frac{\chi }{2}\right)\Delta G_{A}^{0}-\left(\frac{\chi }{2}\right)F\varphi_{f/s}^{0}+\Delta G_{s}^{0}$$

where ω is the energy term related to the energy of absorption of aggressive anions into the oxygen vacancies (J mol^−1^). The more negative the value of ω, the more energetically favorable is the absorption process. Using the slope of the experimental plot in Figure 8 and applying Equation (1), the value of *α* was calculated to be 0.19 [53]. Thus, nineteen percent of any change in the applied voltage appears as a change in the voltage drop across the bl/s interface.

As discussed earlier, according to the PDM, *J_m_* is a crucial parameter governing the breakdown of the barrier layer of a passive film. Due to the absorption of aggressive anions (Cl^−^) into a positively-charged oxygen vacancy in the surface of the barrier layer, through different mechanisms that are proposed by the PDM, cation vacancies at the bl/s interface are generated and migrate toward the metal surface. The competitive rate of cation vacancy migration and annihilation at the m/bl interface determines the dynamics of the breakdown of the barrier layer and meta-stable pit formation, with breakdown occurring when *J_m_* < *J_ca_* [23,66]. One way of estimating *J_m_* is by using the passive current density at the point of breakdown $i_{ss}^{bd}$ (10^−6^ A.cm^−2^) obtained from Figure 7 and substituting it into the following equation [58]:

(4) $$J_{m}\leq \frac{i_{ss}^{bd}N_{v}}{\chi F}$$

Since the passive current is carried by metal interstitials, cation vacancies, and oxygen vacancies, with cation interstitials or oxygen vacancies being the majority point defect, the inequality in Equation (4) appears. Assuming the oxidation state of the aluminum cation within the barrier layer is equal to 3, $J_{m}\leq 2.08\times 10^{12}$ cm^−2^ s^−1^.

The analytical relationship in Equation (1) can be rewritten as [23,56]

(5) $$E_{c}=E_{0}-\frac{\beta }{\alpha }pH-\frac{2.303RT}{\alpha F}log\left(a_{Cl^{-}}\right)$$

where *E*_0_ is the standard breakdown potential, i.e., the breakdown potential for $a_{Cl^{-}}$ = 1 and pH = 0 ($a_{H^{+}}$ = 1). Based on Equation (5) and using the previously-derived value of *α* = 0.19 from Figure 8, *β* is calculated to be −0.014 V. Additionally, by employing the slope and the intercept of the linear fit in Figure 8 and Figure 9 in Equations (5)–(7) were obtained, respectively:

(6) $$E_{c}=-0.77-0.34log\left(a_{Cl^{-}}\right)$$

and

(7) $$E_{c}=-0.92+0.07pH.$$

Considering the values of *α* = 0.19 and *β* = −0.014 V, *E*_0_ was calculated to be −1.27 V and −1.3 V from Equations (6) and (7), respectively. This agreement of the values of *E*_0_ demonstrates the viability of the PDM for describing the passivity breakdown on AA2098-T851 in chloride-containing solutions.

The relationship between *E_c_* and *ν*^1/2^ that is provided by the PDM is shown in Equation (8) [58]:

(8) $$E_{c}\left(\nu \right)={\left(\frac{2\xi RT}{J_{m}\chi \alpha F}\right)}^{\frac{1}{2}}\nu^{\frac{1}{2}}+E_{c}\left(\nu =0\right)$$

where *E_c_* (*υ* = 0) is the breakdown voltage at zero scan rate and *ξ* is the critical vacancy concentration. Applying Equation (8) using the parameters that were previously obtained from experiments (*α* and *J_m_*) and using the gradient of *E_c_* versus *ν*^1/2^ from Figure 10, the value of *ξ* ≤ 5.6 × 10^13^ cm^−2^ was calculated.

The value of *ξ* can also be calculated from the unit cell dimensions of the base metal (Al) and the barrier layer (Al_2_O_3_), assuming that, in order to induce separation of the barrier layer from the substrate, condensation occurs on all cation sublattice sites, or on all metal sites on the substrate lattice at the m/bl interface. The base metal has a face-centered cubic structure with a lattice constant of 0.409 nm [65]. Additionally, depending on the chemical and physical properties of the Al and the environment, the Al_2_O_3_ passive layer could have different forms from the amorphous air-borne film to various crystalline structures [55]. Considering cubic γ-Al_2_O_3_ with the unit cell dimension of 0.79 nm as the Al_2_O_3_ barrier layer crystal structure, in the monolayer of the unit cell of the Al and its barrier layer, the density of Al atoms were calculated to be *ξ* (theoretical) = 2.4 × 10^15^ cm^−2^ and 3.4 × 10^15^ cm^−2^, respectively [65]. These results are in reasonable agreement with the value obtained from PDM and Equation (4) [*ξ* (experimental) = 5.6 × 10^13^ cm^−2^]. A similar finding was previously reported by Fonseca et al. [65], who found a somewhat closer agreement between the values of the calculated and experimentally-derived values of *ξ* than that reported here. However, it must be noted that perhaps it is not necessary that all of the cation sites at the interface be occupied by cation vacancies for the separation of the barrier layer from the metal substrate to occur, in which case *ξ* (experimental) < *ξ* (theoretical).

The reader will note that the fundamental event in passivity breakdown, according to the PDM, is the formation of a cation vacancy condensate at the m/bl interface. The formation of such blisters as the precursors to passivity breakdown on aluminum has been detected and described by Bargeron and Givens and by McCafferty et al., amongst others [68,69,70,71,72,73,74]. Indeed, the present findings are entirely in concert with that previous work and provide an analytical basis for the vacancy condensation mechanism for passivity breakdown.

### 4.2. Cumulative Probabilities of the E_c_

As mentioned previously, *E_c_* is a near-normally distributed function, which is a result of assuming a normal distribution of breakdown sites on the surface with respect to the cation vacancy diffusivity [42,43,58,83,84,85]. Thus, the weak spots in the barrier layer have a high cation vacancy diffusivity with the sites being normally-distributed in that parameter, with the width of the distribution that is defined by the standard deviation, $\sigma_{D}$. A wider distribution of the breakdown sites with respect to *E_c_* is a reflection of a larger $\sigma_{D}$.

**Figure 11:**
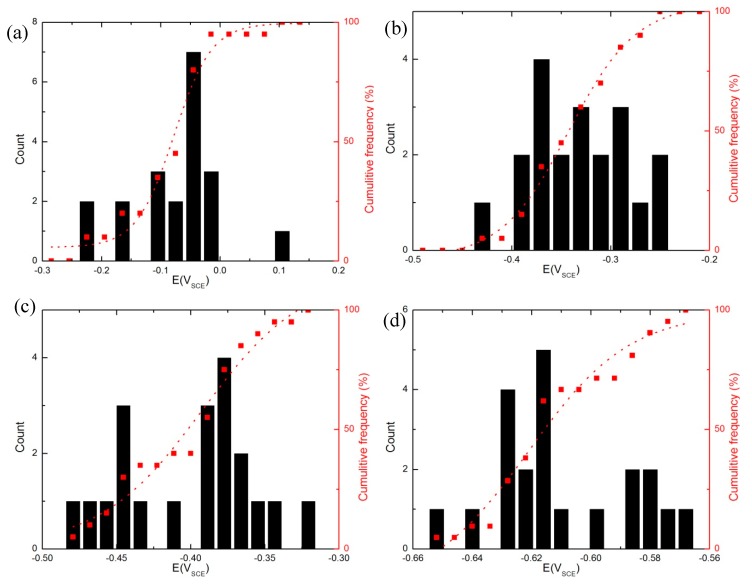
The statistics plot of *E_c_* in 0.1 M NaHCO_3_ buffer solution in a CO_2_ atmosphere at 25 °C, (**a**) 0.01 M NaCl, (**b**) 0.05 M NaCl, (**c**) 0.1 M NaCl and (**d**) 0.5 M NaCl.

Figure 11 shows the cumulative distributions of *E_c_* for Specimen S3 in different chloride ion concentration at 0.166 mV/s in a CO_2_ atmosphere. The near-normal distribution of *E_c_* was indicated by an almost straight line in the cumulative frequency. The distributions varied widely in width and in some cases, such as 0.01 M [Cl^−^], it was as wide as 300 mV, which might be due to the different inclusions and microstructure of AA2098-T851.

To investigate the propensity toward pitting corrosion, the PDM proposes analytical equations for the cumulative probability distribution function in breakdown potential as follows [23,66,86]:

(9) $$\frac{dN}{dE_{c}}=\frac{-\gamma \prime b}{\sqrt{2\pi }\sigma_{D}a_{Cl^{-}}^{\chi /2}}exp\left[\frac{-{\left(e^{-\gamma \prime E_{c}}-e^{-\gamma^{\prime \bar{E_{c}}}}\right)}^{2}b^{2}}{2\sigma_{D}^{2}a_{Cl^{-}}^{\chi }}\right]\;exp\left(-\gamma \prime E_{c}\right)$$

where

(10) $$\gamma^{\prime }=\frac{\chi \alpha F}{2RT}$$

and $\bar{E_{c}}$ is the mean value of the critical breakdown potential and can be calculated by

(11) $$\bar{E_{c}}=\frac{1}{\gamma^{\prime }}ln\left(\frac{b}{\bar{D}}\times a_{Cl^{-}}^{\frac{\chi }{2}}\right).$$

The cumulative probability distribution function in *E_c_* is defined as

(12) $$p\left(E_{c}\right)=100\times \frac{\int_{-\infty }^{E_{c}}\left(\frac{dN}{dE_{c}}\right)dE_{c}}{\int_{-\infty }^{+\infty }\left(\frac{dN}{dE_{c}}\right)dE_{c}}.$$

**Figure 12:**
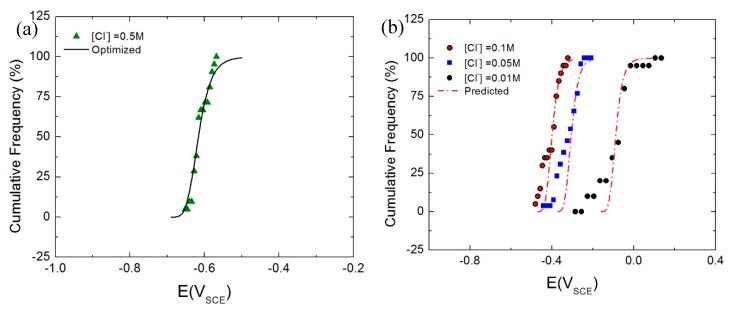
The comparison of the optimized (**a**) and calculated (**b**) cumulative probabilities of *E_c_* from Point Defect Model (PDM) with the experimental data for the AA2098–T851 (S3) in chloride solution with NaHCO_3_ buffer in CO_2_ atmosphere at 25 °C [53].

Therefore, the statistical distribution of *E_c_* for the AA2098-T851 in the chloride solution (Figure 11) using Equation (9), (11) and (12) was interpreted. To do that, experimental results at 0.5 M [Cl^−^] from Figure 11d were optimized using Wolfram Mathematica 8 software. Table 2 shows the parameters that were derived from the optimization, including the mean diffusivity of the cation vacancy ($\bar{D}$ = 3.5 × 10^−18^ cm^2^ s^−1^) and its standard deviation (***σ_D_*** = 0.3 × $\bar{D}$ cm^2^ s^−1^). The cation vacancy diffusivity reflects the accumulated effects of the combination of different populations of breakdown sites (e.g., different precipitates/inclusions, grain boundaries and other microstructural defects at the m/bl interface) that exists on the metal surface –as some were characterized in previous sections– and shown in Figure 12b for the lowest chloride concentrations. It is postulated that differentiation between different populations of breakdown sites is most clearly manifested at less severe breakdown conditions; i.e., at the lowest chloride concentration as shown in Figure 12b. Therefore, the microstructure is included in the PDM indirectly as it is postulated that passivity breakdown occurs at points of high cation vacancy diffusivity, such as at the intersection of the barrier layer with precipitates, at “ghost” grain boundaries, and perhaps at dislocations that project through the barrier layer, for example.

It should be noted that the PDM predicts that those sites that have the most negative breakdown potentials are characterized by the highest cation vacancy diffusivity and vice versa. They are also the sites that have the minimum induction time, thereby tying the microstructure to the statistics of passivity breakdown for the first time. The parameter values used in calculating the cumulative distributions of *E_c_* that were obtained by optimization of Equation (12) on the experimental data for 0.5 M [Cl^−^] (Figure 12a) were further used to predict *E_c_* cumulative distribution at other chloride concentrations (Figure 12b). Figure 12b compares the cumulative probability plots in *E_c_* as a function of chloride concentration from the experiments (solid data points) with the PDM prediction (solid line) calculated using the data in Table 2. The close agreement between the experimental and calculated results provides a strong confirmation of the validity of the PDM for describing the breakdown of passivity on the AA2098-T851. The results obtained from the optimization of the PDM on the distributed breakdown potential data allows one to calculate the breakdown potential and its distribution for any given pH, temperature, and chloride ion concentration. Furthermore, for a more in-depth understanding of the underlying mechanism of the observations made in this study, the passivation and growth behavior of the barrier layer on the surface of the same alloy can also be investigated with the PDM using point defect reaction kinetics derived from EIS analysis which is the topic of the next paper (Part III).

## 5. Summary and Conclusions

In this work, the influence of chloride concentration, pH, and polarization scan rate on passivity breakdown of AA2098-T851 (S3), which was previously shown to exhibit higher pitting potential and corrosion resistance than AA2029-T8 (S1) and AA2060-T8 (S2), was investigated and the findings are summarized as follows:

- The linear dependence of *E_c_* on the logarithm of the chloride activity, on the pH, and on the square root of the voltage scan rate (*ʋ*^1/2^) agree well with the predictions of the PDM.
- From the experimental results, using the PDM, some characteristic parameters, such as (*α*, *β*, *J_m_* and *ξ*) were extracted which were later employed in the optimization of the near-normal distribution of *E_c_*.
- Satisfactory agreement between *ξ* that was obtained from the experiment with that estimated theoretically from the passive layer/metal structure validates the predictions made by the PDM.
- By numerical analysis (optimization) of the theoretical cumulative probability function, as given by the PDM, on the experimental breakdown potential data for a single chloride concentration (0.5 M), values for various model parameters were derived.
- The derived model parameters satisfactorily account for the cumulative probability functions measured at other chloride concentrations.
- The above findings can be used to predict the passivity breakdown and failure of the AA2098-T851 in different pH, [Cl^−^] and temperature and similarly, the methodology could be extended to other alloys as well.

## Figures and Tables

**Table 1:** The nominal chemical compositions (wt %) of the aluminum alloys investigated in this work [26].

Number	Material	Cu	Li	Mg	Mn	Ag	Zr	Zn	Al
2029-T8 (S1)	2029-T8	3.46	-	0.80	0.26	0.04	-	0.01	Bal.
2060-T8 (S2)	2060-T8	3.63	0.78	0.67	0.25	0.04	0.06	0.29	Bal.
2098-T851 (S3)	2098-T851	3.71	1.29	0.26	0.03	0.03	0.06	0.01	Bal.

**Table 2:** The parameters used in the calculation of the cumulative probabilities in breakdown potential for the AA2098-T851 in chloride solution in a CO_2_ atmosphere at 25 °C [53].

Parameter	Definition	Value	Source
*α*	Polarizability of bl/ol Interface	0.19	From Figure 10
*β*	Dependence of the potential drop across bl/ol upon pH	−0.014 V	From Figure 11
*ε*	Electric Field Strength	3 × 10^6^ V cm^−1^	Assumed
*χ*	Oxidation state	3	Assigned
*Ω*	Molar volume of oxide per cation	12.91 cm^3^ mol^−1^	From density
$\bar{D}$	The mean diffusivity of cation vacancy	3.5 × 10^−18^ cm^2^ s^−1^	From fitting
$\sigma_{\bar{D}}$	The standard deviation for $\bar{D}$	1.05 × 10^−18^ cm^2^ s^−1^	From fitting
*ξ*	The critical vacancy concentration	5.6 × 10^13^ cm^−2^	From Figure 12
*J_m_*	The critical vacancy flux	2.08 × 10^12^ cm^−2^ s^−1^	From Figure 9
*ω*	The energy term	−24888 J mol^−1^	From Figure 10
